# Evaluation of the association of *UBASH3A* and *SYNGR1* with rheumatoid arthritis and disease activity and severity in Han Chinese

**DOI:** 10.18632/oncotarget.21875

**Published:** 2017-10-17

**Authors:** Dan Liu, Jiayu Liu, Guofeng Cui, Haojie Yang, Tuanping Cao, Li Wang

**Affiliations:** ^1^ Departement of Rheumatology and Immunology, Xi'an No.5 Hospital, Xi'an, Shaanxi, China; ^2^ Department of Orthopedics, Luoyang Central Hospital Affiliated to Zhengzhou University, Luoyang, Henan, China; ^3^ Department of Prevention and Health Care, Xi’an Jiaotong University Hospital, Xi’an, Shaanxi, China

**Keywords:** rheumatoid arthritis susceptibility, common variants, UBASH3A, SYNGR1, disease activity and severity

## Abstract

Rheumatoid arthritis (RA) is a common complex autoimmune disorder. *UBASH3A* and *SYNGR1* were identified recently as susceptibility genes for RA risk in Korean and European populations, but the genetic aetiology and pathogenesis of RA have not been fully elucidated. We designed a two-stage case-control study including 916 RA patients and 2,266 unrelated healthy controls to identify common genetic variants in *UBASH3A* and *SYNGR1* that predispose Han Chinese individuals to RA. We also evaluated the role of associated variants in clinical manifestations of RA, which may provide clues to the mechanisms involved in the aetiology of RA. We successfully identified two SNPs, rs1893592 in UBASH3A and rs909685 in SYNGR1, as significantly associated with the disease status of RA using our two-stage strategy. The rs1893592 SNP in *UBASH3A* was related with DAS28, CRP level and bone erosion. In summary, our results indicate that genetic variants in *UBASH3A* and *SYNGR1* may modify individual susceptibility to RA in the Han Chinese population and support the role of the *UBASH3A* gene in RA disease activity and severity.

## INTRODUCTION

Rheumatoid arthritis (RA) is a common complex autoimmune disorder that is characterized by erosive symmetrical polyarthritis and systemic extra-articular manifestations [1]. Patients present with chronic synovitis, destruction of joint tissue (cartilage and bone), and impaired joint function [2]. The prevalence of RA is approximately 0.32% (1% in Caucasian populations) in the Chinese population with considerable variation between different populations worldwide [3, 4]. RA is a chronic condition that results in progressive articular damage and functional disability and imposes significant financial and social burdens in patients [5].

The aetiology and pathogenesis of RA have not been fully elucidated, but it may be caused by complex interactions of multiple susceptibility genes, environmental factors, and exposure to certain infections [6–8]. Familiar and twin studies provided robust evidence for the role of genetic factors in the disease [9, 10], and the heritability of RA is estimated at approximately 65% [10]. Genetic association mapping studies, including a genome-wide association study (GWAS), identified a combination of risk alleles from numerous genes that contributed to RA susceptibility [11], and it is becoming evident that RA shares common genetic causative factors in different ethnic populations [12]. More than 100 susceptible loci are associated with RA [13]. Genetic association analyses provide a promising approach to the genetics of RA, but current results account for only a small percentage of the estimated heritability with a lack of systematic biological interpretation [8]. The identification of specific susceptibilities with small effects on RA risk would help reveal the underlying pathological mechanisms of RA and aid in drug discovery and the optimal implementation of established therapies through a greater understanding of disease heterogeneity. Follow-up studies are essential to confirm the GWAS findings and extend these findings to different populations.

Two new RA susceptibility genes (*UBASH3A* and *SYNGR1*) were identified recently using a genome-wide meta-analysis in Korean and European populations [14]. The underlying biological mechanisms of RA are largely unknown, and the contribution of these two genes to RA has not been fully elucidated, despite evidence of strongly significant associations within the Korean and European populations. Therefore, it is necessary to re-evaluate the transferability of the discovered RA susceptibility loci in different ethnic populations to improve our current understanding of the role of these two genes in RA predisposition and help define the primary set of risk alleles for RA susceptibility. The role of these two genes in RA susceptibility in the Han Chinese population has not been assessed previously. Therefore, we designed a two-stage case-control study to further assess the relationship of common variants in these two genes with the increased risk of RA in Han Chinese individuals. There are no reports on the association between these two genes and clinical manifestations of RA, such as number of tender joints (NTJ), number of swollen joints (NSJ) and 28-joint disease activity score (DAS28). The second aim of the present study was to evaluate the role of associated variants with clinical manifestations of RA, which may provide clues to the mechanisms involved in the aetiology of RA.

## RESULTS

### Characteristics of the subjects

A two-stage approach was used in our study, which included 916 RA patients and 2,266 healthy controls in both stages. RA cases and healthy controls were matched by mean age in both stages, and there were no significant differences in gender between cases and controls (Table 1). Table 1 presents demographic and clinical data of the patients.

**Table 1:** The serological and clinic characteristics of RA in subjects

Characteristics	Cases (*N* = 331)	Controls (*N* = 974)		*P*-value (χ^2^ or t)	Cases (*N* = 585)	Controls (*N* = 1,292)	*P*-value (χ^2^ or t)
	Discovery stage				Replication stage		
Age (years)	52.38 ± 8.61		52.14 ± 8.68	0.669 (-0.442)	49.86 ± 7.13	50.24 ± 7.14	0.285 (1.069)
Gender (female/male) (%)	272/59 (82.2%/17.8%)		798/176 (81.9%/18.1%)	0.920 (0.010)	479/106 (81.9%/18.1%)	1059/233 (82.0%/18.0%)	0.964 (0.002)
Disease duration (months)	53.64 ± 27.89		NA	NA	50.28 ± 24.76	NA	NA
RF seropositivity [*n* (%)]	276(83.38%)		NA	NA	469(80.17%)	NA	NA
Anti-CCP positive [*n* (%)]	249(75.23%)		NA	NA	420(71.79%)	NA	NA
Anti-GPI positive [*n* (%)]	214(64.65%)		NA	NA	357(61.02%)	NA	NA
ESR (mm/h)	31.30 ± 5.25		NA	NA	29.06 ± 4.76	NA	NA
CRP (mg/L)	52.82 ± 16.38		NA	NA	46.08 ± 14.49	NA	NA
Tender joint number	8.68 ± 3.72		NA	NA	8.27 ± 3.71	NA	NA
Swollen joint number	3.30 ± 1.73		NA	NA	1.99 ± 1.49	NA	NA
DAS28 score	5.00 ± 0.62		NA	NA	4.75 ± 0.64	NA	NA
Patients with erosions [*n* (%)]	157(47.43%)		NA	NA	298(50.94%)	NA	NA
Patients with extra-articular features [*n* (%)]	47(14.20%)		NA	NA	73(12.48%)	NA	NA

Data were shown as mean ± SD (standard deviation), except gender. NA, not applicable; RA, rheumatoid arthritis; RF, rheumatoid factor; anti-CCP, anti-cyclic citrullinated peptide antibody; anti-GPI, anti-Glucose phosphate isomerase; ESR, erythrocyte sedimentation rate; CRP, C-reactive protein; DAS28, 28-joints disease activity score.

### Single marker-based association of selected SNPs with RA risk

Two SNPs, rs1893592 (*UBASH3A*) and rs909685 (*SYNGR1*), were nominally significant in all three genetic models. These two SNPs were tested in the validation stage, and the *P* value threshold used in this stage was 0.05/2 = 0.025, and the two significant SNPs in the discovery stage were successfully validated. Table 2 summarizes the significant results of single marker-based analyses. The tested alleles for the rs1893592 and rs909685 SNPs exhibited protective effects with a relative risk of 0.6-0.7. Supplementary Tables 3–8 summarize the full results of single marker-based analyses.

**Table 2:** Significant results of single marker based association analyses

Chr	SNP	Position	A1	OR_1^*^	*P*-value_1^*^	OR_2^**^	*P*-value _2^**^	Combined OR^***^	Combine *P*-value ^***^
21	rs1893592	42434957	C	0.6392	3.26 × 10^-5^	0.7027	2.49 × 10^-5^	0.6791	4.50 × 10^-9^
22	rs909685	39351666	T	0.6809	0.0006	0.7202	0.0002	0.7043	2.01 × 10^-5^

Chr, Chromosome; A1, minor allele for odds ratio; OR, odds ratio; odds ratio.

^*^The odds ratio and *P* value of the discovery stage in additive model.

^**^ The odds ratio and *P* value of the replication stage in additive model.

^***^The odds ratio and *P* value of the combined sample in additive model.

### Bioinformatics analyses and functional prediction of significant SNPs

Genes that interacted with *UBASH3A* and *SYNGR1* were identified using STRING (Supplementary Figure 1). We also examined the potential functional significance of these two susceptible SNPs. Neither rs1893592 nor rs909685 were located in exon regions, which suggests indirect effects on the protein coding process. We examined both SNPs in RegulomeDB to investigate their potential biological significance. Rs909685 had a score of 1b, which indicates that this SNP locates within a very important functional region (including eQTL, TF binding, any motif, DNase footprint and DNase peak). However, there was no data for rs1893592 in RegulomeDB.

### Analyses for the effect of significant SNPs on disease activity and RA severity

Associations between the two significant SNPs, rs1893592 and rs909685, and the four disease activity/severity related measures of RA (DAS28, CRP level, extra-articular and bone erosion) were analysed using all of our 916 RA patients. Table 3 summarizes the results. Rs909685 was not significantly associated with any of the four measures, but rs1893592, which is located in the UBASH3A gene, was significantly associated with 3 of the 4 measures (DAS28, CRP level and bone erosion).

**Table 3:** Results of association study between the four disease activity/severity related measures of RA and two identified susceptible SNPs

					Discovery Stage			Replication Stage			Combined		
Clinical Measurements	Chr	SNP	Position	A1	Beta/OR	STAT	*P*-value	Beta/OR	STAT	*P*-value	Beta/OR	STAT	*P*-value
DAS28^*^	21	rs1893592	42434957	C	-0.72	-17.46	1.28 × 10^-48^	-0.63	-17.19	7.70 × 10^-54^	-0.67	-23.92	1.83 × 10^-98^
	22	rs909685	39351666	T	0.10	1.66	0.0990	0.02	0.44	0.6621	0.04	1.15	0.2508
CPR level^*^	21	rs1893592	42434957	C	-8.95	-6.05	4.02 × 10^-9^	-4.07	-3.96	8.43 × 10^-95^	-5.8	-6.84	1.48 × 10^-11^
	22	rs909685	39351666	T	1.85	1.10	0.2729	-0.28	-0.26	0.7943	0.39	0.43	0.6647
Extra-Articular^**^	21	rs1893592	42434957	C	1.13	0.44	0.6620	1.19	0.81	0.4193	1.16	0.87	0.3822
	22	rs909685	39351666	T	1.16	0.50	0.6177	1.34	1.38	0.1687	1.28	1.45	0.1476
Erosion^**^	21	rs1893592	42434957	C	0.47	-3.63	0.0003	0.59	-3.57	0.0004	0.55	-4.91	8.95 × 10^-7^
	22	rs909685	39351666	T	0.90	-0.49	0.6239	1.36	2.05	0.0400	1.19	1.46	0.1433

Chr, Chromosome; A1, minor allele; CRP, C-reactive protein; DAS28, 28-joints disease activity score; OR, odds ratio.

^*^ linear regression models were fitted.

^**^ logistic regression models were utilized.

## DISCUSSION

Genetic association analyses are a promising approach to the genetics of complex diseases, such as schizophrenia [15–20], but current results account for only a small percentage of the estimated heritability with a lack of the corresponding biological interpretation [21]. Genetic heterogeneity exists in different populations, and we designed a two-stage case-control study to confirm and extend previous findings. To the best of our knowledge, this report is the first association study of common variants in *UBASH3A* and *SYNGR1* with RA risk and disease activity and severity in a Han Chinese population. Several previous studies have examined these two loci. In a 2013 published genome-wide meta analysis based on European and Asian (Japanese) cohorts [22]. The two SNPs, rs1893592 and rs909685, were both identified to be significantly associated with RA in European cohorts (*P* = 9.8×10^–9^ and 6.4×10^–12^). However, they failed to pass the genome-wide threshold in Japanese cohorts (*P* = 1.3×10^–4^ and 2.0×10^–7^). Another GWAS published in 2014 based on Chinese population did not report the two loci to be significantly associated with RA risk, although it expanded the list of alleles that confer risk of RA [23]. *UBASH3A* and *SYNGR1* were significantly associated with RA in a recent meta-analysis of Korean and European populations. This study also identified rs1893592 and rs909685 as significant SNPs [14]. The present study successfully replicated these previous findings in a Han Chinese population. A major reason for the disparities between our study and the 2014 GWAS conducted by Jiang *et al.* [23] might be the difference of study design between GWASs and candidate genes based analyses. A typical *P* value threshold used in GWASs is 5×10^–8^. Bonferroni correction can successfully control type I error brought by multiple comparisons at the cost of dropping many true hits which failed to be identified simply because of not reaching this super conservative *P* value threshold. Notably, the ORs of the tested alleles for rs1893592 and rs909685 were 0.8–0.9 in the meta-analysis of Korean and European populations, and the ORs in our study were slightly lower (0.6–0.7). This difference may be explained by potential genetic heterogeneity between different populations. Our study also tested the potential connection between these significant SNPs and disease activity/severity related measures of RA. The rs1893592 SNP (*UBASH3A*) was significantly associated with 3 of the 4 disease measures (DAS28, CRP level and bone erosion). The C allele (tested allele) of rs1893592 exhibited a pattern of negative correlation with disease activity and severity in RA patients. Our findings indicated that the C allele of rs1893592 may lower the odds of RA onset in the general population, but this allele indicates a reduction in disease severity in RA patients.

Previous studies suggested a key role for T-cell activation pathways in the initiation and/or perpetuation of RA [24]. Biomedical research identified that *UBASH3A* encodes one of the two family members of the T-cell ubiquitin ligand (TULA) family. This protein facilitates growth factor withdrawal-induced apoptosis in T cells [25]. These previous studies hint at the biomedical basis of our results of an association between *UBASH3A* and RA activity and severity. We only know that rs189359 is a common variant located in the intronic region of *UBASH3A* because RegulomeDB had no data to annotate this SNP. Therefore, we cannot establish whether the effect detected on this SNP was due to its potential biological significance. *SYNGR1*, unlike *UBASH3A*, does not encode a protein that is related to the human immune system. This gene encodes an integral membrane protein that is associated with presynaptic vesicles in neuronal cells [26]. Research using single and double knockout mutant mice indicates that the absence of Syngr1 does not alter the architecture or composition of synapses in the brain, but electrophysiological recordings demonstrated a severe reduction of the short- and long-term synaptic plasticity in the CA1 hippocampus in gene knockout mice [26]. *SYNGR1* was not linked to any major complex disorders. Our bioinformatics analysis revealed that rs909685 had a RegulomeDB score of 1b, which indicates that this SNP has a very significant function. This finding suggests that this SNP has functional significance, but there is no experimental evidence to validate this hypothesis. Future functional studies are warranted.

There are several advantages of this study. Analyses of only some SNPs are not sufficient to draw conclusions [27–29]. Our genetic association study examined the relationship between RA status and the candidate genes and the association between significant SNPs and disease activity/severity related measures. Therefore, our study replicated previous studies in a Han Chinese population and further demonstrated the potential association between these genes and some clinical measures of RA to characterise the underlying mechanisms of disease initiation and progression. The two-stage study design used in our study provided sufficient statistical power and reduced the experimental cost to a bearable level. However, our study also suffered from several limitations. Firstly, we do not perform any procedures to adjust for population stratification in our statistical analyses. This fact may be problematic, especially because the Han Chinese population is one of the most genetically diverse ethnic groups in the world. Our candidate gene-based study makes it difficult for us to perform confounding control procedures, such as principle component analysis, which requires large numbers of SNPs. However, we tried our best to at least partially control this potential confounding factor. Geographic region is a good proxy for genetic matching in the Han Chinese population [30, 31]. Our recruitment process only included study subjects with no migration history within the previous three generations. We believe this procedure may at least partially guarantee the purity of the genetic background of our study subjects. In addition, most of the disease history provided by our study subjects was based on our inquiry from the patients due to lack of previous clinical records. The information obtained might be inaccurate. Secondly, hospital-based case-control studies are always problematic, leading to selection sample bias, although it is difficult for us to validate this bias in the study. Another limitation of this study is that the residual of the linear models are not distributed normally (Supplementary Figure 2) for RA severity related indicators (DAS28 and CRP), which indicates that some underlying affective variables were failed to be detected and adjusted in our statistical model. Although the effects of these variables were limited according to the residual plot, it is still worrisome and need to be carefully examined in the future study. We also only considered common SNPs and omitted rare variants. A sequencing-based study may provide more information than our study because of the unknown functional significance of the rs1893592 SNP. In this study, most of our study subjects were RA patients with low or no disease modifying antirheumatic drugs despite long disease duration due to their low socioeconomic status. Therefore, it needs to be careful when applying the results of our study to RA patients of other populations who received early treatment.

In conclusion, this study replicated previous findings on susceptibility of *UBASH3A* and *SYNGR1* to RA in a Han Chinese population. We identified two SNPs, rs1893592 in *UBASH3A* and rs909685 in *SYNGR1*, as significantly associated with RA status. We also examined the association between these two significant SNPs and 4 disease activity/severity related measures of RA. The rs1893592 SNP of *UBASH3A* was related with 3 of the 4 RA measures. Future studies should replicate our significant findings on the clinical measures of RA in other ethnic groups.

## MATERIALS AND METHODS

### Subjects and clinical assessments

Two separate datasets were included in this study. Subjects in the discovery dataset included 331 RA patients (aged 36–65 years) and 974 healthy age-matched controls (aged 36–65 years) who were recruited from Luoyang Central Hospital Affiliated to Zhengzhou University between January 2013 and March 2016. Subjects in the replication dataset consisted of 585 RA patients (aged 38–62 years) and 1,292 healthy age-matched controls (aged 38–62 years) who were enrolled from the Fifth Hospital of Xi‘an between January 2014 and February 2016. The two-stage subjects recruited in this study were random genetically unrelated Han Chinese individuals from the city of Luoyang in Henan Province and Xi’an in Shaanxi Province, with no migration history within the previous three generations. In each stage, the patients lived in the same geographical region as the controls. All patients with RA were diagnosed according to the 2008 Classification Criteria of the American College of Rheumatology, and all controls had no history of infectious, rheumatism or chronic inflammatory autoimmune diseases. In both stage, the controls were randomly enrolled from unrelated healthy individuals under routine health screening in the health check-up centers of these hospitals based on the selection criteria of frequency-matched age (±5 years) and gender of the patients. This study was performed in accordance with the ethical guidelines of the Helsinki Declaration of 1975 (revised in 2008) and was approved through the Local Ethics Committees of the hospitals. Informed consent was obtained from all subjects.

All RA patients provided disease history, especially for presenting symptoms, joint affection and extra-articular features, and medication history. Disease activity was measured using the DAS28, which is calculated from the 28 tender and 28 swollen joint counts, and erythrocyte sedimentation rate (ESR) [32]. No RA patients were treated with intra-articular corticosteroids, MTX or biological agents at the time of disease activity assessment. X-rays of the hands and feet were obtained for all RA patients, and a rheumatologist and radiologist evaluated joint erosion. We noted only the presence or absence of erosion without calculation of radiological scores. Patients were considered to have an erosive RA if one or more definitive erosions were apparent in any of the peripheral joints that are predictive of disease progression [33]. Patients were divided into two groups, erosive RA and non-erosive RA. Patients were also grouped according to the presence or history of extra-articular features. Laboratory parameters were recorded, including rheumatoid factor (RF), anti-cyclic citrullinated peptide antibody (aCCP), anti-glucose phosphate isomerase (aGPI), ESR and C-reactive protein (CRP). Demographic information was collected from each patient at the time of study enrolment.

### SNP selection and genotyping

Firstly, we searched for all SNPs with minor allele frequencies (MAF) ≥ 0.01 between 10 kb upstream and 10 kb downstream of *UBASH3A* and *SYNGR1* genes in the HapMap HCB database. MAF ≥ 0.01 with pair-wise tagging and r^2^ ≥ 0.9 were used as the cut-off criteria during tag SNP selection, which resulted in 18 and 11 tag SNPs covering the regions of *UBASH3A* and *SYNGR1*, respectively, for our study. We also selected a significantly associated SNP (rs909685) in *SYNGR1* from a previous GWAS report [14]. The above criteria identified 30 tag SNPs for further analyses (Supplementary Table 1).

Peripheral blood was drawn from a vein into a sterile tube containing ethylenediamine tetraacetic acid (EDTA). Genomic DNA was extracted from peripheral blood leukocytes according to the manufacturer’s protocol (Genomic DNA kit, Axygen Scientific Inc., California, USA). DNA was stored at -80°C for SNP analysis. Genotyping was performed for all SNPs using the MassARRAY platform (Sequenom, San Diego, California, USA). Briefly, SNPs were genotyped using high-throughput, matrix-assisted laser desorption ionization–time-of-flight (MALDI–TOF) mass spectrometry, and the resulting spectra were processed using Typer Analyzer software (Sequenom, San Diego, California, USA) [34]. Case and control status were blinded during all genotyping processes for quality control. Five percent of random samples were repeated, and the results were 100% concordant.

### Statistical and power analyses

#### Power analyses

We conducted a comprehensive statistical power analyses with combinations of multiple risk allele frequencies and relative risks using R package gap (https://cran.r-project.org/web/packages/gap/) [35] to evaluate the statistical power of our two-stage study design. The statistical power was estimated under additive model. The joint statistical powers of the discovery and validation stages are summarized in Supplementary Table 2. Our study design and sample size can achieve 80% statistical power to detect a risk allele with minor allele frequency (MAF) ≥ 0.3 and relative risk ≥ 1.3 or ≤ 0.8 (Supplementary Table 2).

### Single marker-based association of selected SNPs with RA risk

Logistic regression models were fitted to evaluate the potential genetic associations between selected SNPs and RA risk. Three genetic models were analysed: additive, dominant and recessive. Age and gender were included as two covariates for each logistic regression model to adjust the potential confounding effects. A two-stage study design was used in our analyses. We conducted analyses on all 30 selected SNPs in the discovery stage. SNPs with *P* values that passed the threshold of nominal significance (< 0.05) were tested in the validation stage. In addition, we have also analyzed combined sample by the fitting logistic model. Besides age and gender, cohort was also included as a covariate in the logistic model. Bonferroni correction was used for the validation stage to address the multiple comparison problem. The genetic association analysis software Plink [36] was used for the analyses described above.

### Bioinformatics analyses and functional prediction of significant SNPs

We used the protein-protein interaction database STRING (http://string-db.org/) [37] to identify genes that interacted with our candidate genes. We also investigated the potential functional significance of the identified candidate SNPs using RegulomeDB (http://www.regulomedb.org/) [38].

### Analyses for the effect of significant SNPs on disease activity and RA severity

We chose four disease activity/severity-related measures of RA (DAS28, CRP level, extra-articular manifestations and bone erosion) for further analyses to investigate the potential effects of disease-susceptible SNPs and RA severity and activity. Linear regression models were fitted for quantitative measures (DAS28 and CRP level), and logistic regression models were used for qualitative measures (extra-articular and bone erosion). Gender, age, cohorts, and presence of RF and CCP were included in linear and logistic models as covariates to adjust the potential confounding effects.

## SUPPLEMENTARY MATERIALS FIGURES AND TABLES


