# Supplementary material for: Evaluation of the association of UBASH3A and SYNGR1 with rheumatoid arthritis and disease activity and severity in Han Chinese
Source: Oncotarget. 2017 Oct 17;8(61):103385–92. doi: 10.18632/oncotarget.21875 (PMC5732735; doi:10.18632/oncotarget.21875)
Supplement: Supplementary file 1 [file oncotarget-08-103385-s001.pdf]

Evaluation of the association of *UBASH3A* and *SYNGR1* with rheumatoid arthritis and disease activity and severity in Han Chinese

SUPPLEMENTARY MATERIALS

Supplementary Table 1: Information of the 30 selected SNPs tested in the discovery stage

Chr	Position	SNP	Allele	Function	Gene	MAF	HWE
21	42416077	rs11203203	A/G	intron,untranslated-5	*UBASH3A*	0.0881	0.8447
21	42415746	rs11700858	A/C	intron,untranslated-5	*UBASH3A*	0.0858	0.6855
21	42431928	rs12482947	C/T	intron	*UBASH3A*	0.4494	0.9484
21	42418534	rs13048049	A/G/T	ncRNA,missense	*UBASH3A*	0.0766	0.5021
21	42432123	rs13052676	C/T	coding-synon,ncRNA	*UBASH3A*	0.2628	0.8683
21	42434957	rs1893592	A/C	intron,ncRNA	*UBASH3A*	0.2759	0.7593
21	42438382	rs1893593	A/G	intron,untranslated-3	*UBASH3A*	0.3107	1
21	42442356	rs2254368	A/G	intron	*UBASH3A*	0.4356	1
21	42404027	rs2277800	C/T	ncRNA,missense,untranslated-5	*UBASH3A*	0.1287	1
21	42426620	rs2839509	A/G	intron	*UBASH3A*	0.1552	1
21	42447734	rs2839519	A/G	near-gene-3	*UBASH3A*	0.1406	1
21	42406235	rs3746923	C/T	intron	*UBASH3A*	0.4073	0.9473
21	42406509	rs3746925	C/T	intron	*UBASH3A*	0.2375	1
21	42446949	rs3827232	C/T	intron	*UBASH3A*	0.1966	0.9196
21	42435358	rs4101	C/T	intron,ncRNA	*UBASH3A*	0.2268	0.9267
21	42405366	rs7282058	C/T	intron	*UBASH3A*	0.3107	0.9405
21	42446395	rs884339	A/G	intron	*UBASH3A*	0.3498	0.9436
21	42416281	rs9976767	A/G	intron,untranslated-5	*UBASH3A*	0.2770	0.9358
22	39350419	rs12166817	A/C	intron	*SYNGR1*	0.1046	1
22	39342420	rs137682	A/C	unknown	*SYNGR1*	0.1134	1
22	39342720	rs137684	C/T	unknown	*SYNGR1*	0.1061	0.8657
22	39343623	rs137685	C/T	unknown	*SYNGR1*	0.0969	0.8555
22	39373813	rs1569499	C/T	intron	*SYNGR1*	0.0682	0.4557
22	39365250	rs2012907	A/G	intron	*SYNGR1*	0.0881	0.4191
22	39360849	rs2049986	C/G	intron	*SYNGR1*	0.2057	1
22	39351775	rs2069235	A/G	intron	*SYNGR1*	0.2816	0.9372
22	39371006	rs5750806	A/G	intron	*SYNGR1*	0.0732	0.3503
22	39393081	rs5995729	A/C	unknown	*SYNGR1*	0.0759	1
22	39364878	rs739363	G/T	intron	*SYNGR1*	0.0851	0.5302
22	39351666	rs909685	A/T	intron	*SYNGR1*	0.2398	0.5028

Chr, Chromosome; MAF, minor allele frequency; HWE, Hardy-Weinberg equilibrium.

Supplementary Table 2: Summarized results of the comprehensive statistical power analyses for the two-stage study design

MAF<br>Relative Risk	0.05	0.1	0.15	0.2	0.25	0.3	0.35	0.4	0.45
0.7	**0.84**	**0.982**	**0.998**	**1**	**1**	**1**	**1**	**1**	**1**
0.8	0.443	0.703	**0.841**	**0.91**	**0.946**	**0.964**	**0.974**	**0.979**	**0.981**
0.9	0.135	0.213	0.281	0.339	0.385	0.422	0.449	0.468	0.478
1.1	0.007	0.014	0.022	0.029	0.036	0.041	0.046	0.048	0.050
1.2	0.035	0.087	0.144	0.195	0.236	0.268	0.290	0.302	0.306
1.3	0.106	0.259	0.388	0.482	0.546	0.587	0.612	0.623	0.623
1.4	0.226	0.477	0.632	0.722	0.774	**0.805**	**0.821**	**0.826**	**0.823**
1.5	0.375	0.666	**0.801**	**0.867**	**0.901**	**0.918**	**0.926**	**0.928**	**0.925**

MAF, minor allele frequency.

Supplementary Table 3: Full results of the single marker based analyses in discovery stage using additive model

Chr	SNP	Position	A1	OR	L95	U95	STAT	*P*-value
21	rs2277800	42404027	T	1.13	0.87	1.46	0.91	0.3609
21	rs7282058	42405366	T	0.98	0.81	1.19	−0.17	0.8632
21	rs3746923	42406235	T	0.94	0.78	1.12	−0.69	0.4883
21	rs3746925	42406509	T	1.04	0.85	1.28	0.38	0.7027
21	rs11700858	42415746	C	0.98	0.71	1.34	−0.14	0.8887
21	rs11203203	42416077	A	0.94	0.69	1.29	−0.36	0.7207
21	rs9976767	42416281	G	1.08	0.89	1.31	0.78	0.4369
21	rs13048049	42418534	A	0.98	0.71	1.37	−0.11	0.9159
21	rs2839509	42426620	G	1.05	0.82	1.34	0.40	0.6923
21	rs12482947	42431928	T	0.96	0.80	1.14	−0.50	0.6152
21	rs13052676	42432123	T	1.04	0.85	1.27	0.39	0.6972
21	rs1893592	42434957	C	0.64	0.52	0.79	−4.15	3.26E–05
21	rs4101	42435358	C	1.06	0.86	1.31	0.53	0.5963
21	rs1893593	42438382	A	0.97	0.80	1.17	−0.35	0.7246
21	rs2254368	42442356	G	1.03	0.86	1.23	0.35	0.7288
21	rs884339	42446395	A	1.05	0.87	1.26	0.53	0.5960
21	rs3827232	42446949	T	0.96	0.77	1.21	−0.32	0.7497
21	rs2839519	42447734	A	1.15	0.89	1.47	1.06	0.2883
22	rs137682	39342420	A	0.92	0.69	1.22	−0.58	0.5591
22	rs137684	39342720	C	1.04	0.78	1.38	0.26	0.7962
22	rs137685	39343623	T	0.95	0.70	1.29	−0.33	0.7443
22	rs12166817	39350419	A	1.06	0.80	1.41	0.41	0.6799
22	rs909685	39351666	T	0.68	0.55	0.85	−3.42	6.23E–04
22	rs2069235	39351775	G	0.96	0.78	1.16	−0.45	0.6493
22	rs2049986	39360849	G	1.06	0.85	1.32	0.52	0.6023
22	rs739363	39364878	C	1.10	0.81	1.49	0.58	0.5624
22	rs2012907	39365250	G	1.09	0.81	1.48	0.58	0.5650
22	rs5750806	39371006	A	0.99	0.71	1.38	−0.06	0.9547
22	rs1569499	39373813	T	0.94	0.66	1.33	−0.37	0.7146
22	rs5995729	39393081	C	1.05	0.75	1.46	0.29	0.7690

Chr, Chromosome; A1, minor allele for odds ratio; OR, odds ratio; odds ratio; L95, lower bond of 95% confidence interval; U95, upper bond of 95% confidence interval; STAT, statistics

Supplementary Table 4: Full results of the single marker based analyses in discovery stage using dominant model

Chr	SNP	Position	A1	OR	L95	U95	STAT	*P*-value
21	rs2277800	42404027	T	1.14	0.86	1.52	0.90	0.3657
21	rs7282058	42405366	T	0.98	0.76	1.25	−0.20	0.8432
21	rs3746923	42406235	T	0.90	0.69	1.16	−0.84	0.4031
21	rs3746925	42406509	T	1.04	0.81	1.34	0.31	0.7575
21	rs11700858	42415746	C	0.97	0.69	1.36	−0.19	0.8524
21	rs11203203	42416077	A	0.93	0.66	1.31	−0.42	0.6761
21	rs9976767	42416281	G	1.09	0.85	1.40	0.67	0.5053
21	rs13048049	42418534	A	0.96	0.67	1.38	−0.21	0.8373
21	rs2839509	42426620	G	1.05	0.79	1.38	0.32	0.7467
21	rs12482947	42431928	T	0.93	0.71	1.21	−0.55	0.5822
21	rs13052676	42432123	T	1.03	0.80	1.32	0.21	0.8300
21	rs1893592	42434957	C	0.61	0.47	0.79	−3.79	0.0001
21	rs4101	42435358	C	1.08	0.84	1.39	0.57	0.5677
21	rs1893593	42438382	A	0.96	0.75	1.23	−0.34	0.7339
21	rs2254368	42442356	G	1.06	0.81	1.39	0.43	0.6679
21	rs884339	42446395	A	1.07	0.83	1.38	0.53	0.5951
21	rs3827232	42446949	T	0.97	0.75	1.26	−0.23	0.8206
21	rs2839519	42447734	A	1.15	0.87	1.52	0.97	0.3312
22	rs137682	39342420	A	0.91	0.67	1.24	−0.59	0.5547
22	rs137684	39342720	C	1.06	0.78	1.44	0.36	0.7162
22	rs137685	39343623	T	0.97	0.70	1.34	−0.18	0.8589
22	rs12166817	39350419	A	1.06	0.78	1.45	0.38	0.7040
22	rs909685	39351666	T	0.70	0.54	0.90	−2.76	0.0057
22	rs2069235	39351775	G	0.94	0.73	1.21	−0.49	0.6211
22	rs2049986	39360849	G	1.07	0.82	1.38	0.48	0.6304
22	rs739363	39364878	C	1.08	0.77	1.52	0.47	0.6395
22	rs2012907	39365250	G	1.09	0.78	1.52	0.51	0.6082
22	rs5750806	39371006	A	0.97	0.68	1.40	−0.15	0.8806
22	rs1569499	39373813	T	0.93	0.64	1.35	−0.38	0.7007
22	rs5995729	39393081	C	1.05	0.74	1.49	0.27	0.7865

Chr, Chromosome; A1, minor allele for odds ratio; OR, odds ratio; odds ratio; L95, lower bond of 95% confidence interval; U95, upper bond of 95% confidence interval; STAT, statistics.

Supplementary Table 5: Full results of the single marker based analyses in discovery stage using recessive model

Chr	SNP	Position	A1	OR	L95	U95	STAT	*P*-value
21	rs2277800	42404027	T	1.19	0.46	3.09	0.36	0.7224
21	rs7282058	42405366	T	0.99	0.65	1.51	−0.05	0.9618
21	rs3746923	42406235	T	0.96	0.69	1.35	−0.22	0.8235
21	rs3746925	42406509	T	1.10	0.65	1.86	0.34	0.7373
21	rs11700858	42415746	C	1.10	0.29	4.21	0.14	0.8864
21	rs11203203	42416077	A	1.10	0.29	4.16	0.13	0.8944
21	rs9976767	42416281	G	1.15	0.73	1.81	0.60	0.5489
21	rs13048049	42418534	A	1.30	0.33	5.06	0.37	0.7103
21	rs2839509	42426620	G	1.16	0.53	2.53	0.37	0.7124
21	rs12482947	42431928	T	0.96	0.70	1.31	−0.25	0.8005
21	rs13052676	42432123	T	1.14	0.71	1.84	0.53	0.5942
21	rs1893592	42434957	C	0.44	0.25	0.79	−2.75	0.0059
21	rs4101	42435358	C	1.04	0.59	1.84	0.15	0.8833
21	rs1893593	42438382	A	0.96	0.62	1.46	−0.21	0.8368
21	rs2254368	42442356	G	1.02	0.74	1.40	0.11	0.9130
21	rs884339	42446395	A	1.06	0.73	1.54	0.29	0.7719
21	rs3827232	42446949	T	0.88	0.44	1.75	−0.37	0.7120
21	rs2839519	42447734	A	1.34	0.58	3.12	0.68	0.4979
22	rs137682	39342420	A	0.90	0.29	2.79	−0.18	0.8600
22	rs137684	39342720	C	0.81	0.22	2.91	−0.33	0.7435
22	rs137685	39343623	T	0.58	0.13	2.67	−0.70	0.4866
22	rs12166817	39350419	A	1.17	0.36	3.75	0.26	0.7942
22	rs909685	39351666	T	0.33	0.16	0.70	−2.91	0.0036
22	rs2069235	39351775	G	0.96	0.61	1.53	−0.16	0.8744
22	rs2049986	39360849	G	1.11	0.60	2.03	0.33	0.7445
22	rs739363	39364878	C	1.46	0.44	4.90	0.62	0.5369
22	rs2012907	39365250	G	1.30	0.40	4.25	0.43	0.6657
22	rs5750806	39371006	A	1.28	0.33	4.96	0.35	0.7256
22	rs1569499	39373813	T	0.97	0.20	4.86	−0.03	0.9742
22	rs5995729	39393081	C	1.17	0.23	6.09	0.19	0.8492

Chr, Chromosome; A1, minor allele for odds ratio; OR, odds ratio; odds ratio; L95, lower bond of 95% confidence interval; U95, upper bond of 95% confidence interval; STAT, statistics.

Supplementary Table 6: Full results of the single marker based analyses in validation stage using additive model

Chr	SNP	Position	A1	OR	L95	U95	STAT	*P*-value
21	rs1893592	42434957	C	0.70	0.60	0.83	−4.22	2.49E–05
22	rs909685	39351666	T	0.72	0.61	0.85	−3.78	1.55E–04

Chr, Chromosome; A1, minor allele for odds ratio; OR, odds ratio; odds ratio; L95, lower bond of 95% confidence interval; U95, upper bond of 95% confidence interval; STAT, statistics.

Supplementary Table 7: Full results of the single marker based analyses in validation stage using dominant model

Chr	SNP	Position	A1	OR	L95	U95	STAT	*P*-value
21	rs1893592	42434957	C	0.68	0.56	0.83	−3.82	0.0001
22	rs909685	39351666	T	0.69	0.56	0.84	−3.64	0.0003

Chr, Chromosome; A1, minor allele for odds ratio; OR, odds ratio; odds ratio; L95, lower bond of 95% confidence interval; U95, upper bond of 95% confidence interval; STAT, statistics.

Supplementary Table 8: Full results of the single marker based analyses in validation stage using recessive model.

Chr	SNP	Position	A1	OR	L95	U95	STAT	*P*-value
21	rs1893592	42434957	C	0.53	0.34	0.82	−2.84	0.0046
22	rs909685	39351666	T	0.60	0.38	0.97	−2.09	0.0366

Chr, Chromosome; A1, minor allele for odds ratio; OR, odds ratio; odds ratio; L95, lower bond of 95% confidence interval; U95, upper bond of 95% confidence interval; STAT, statistics.

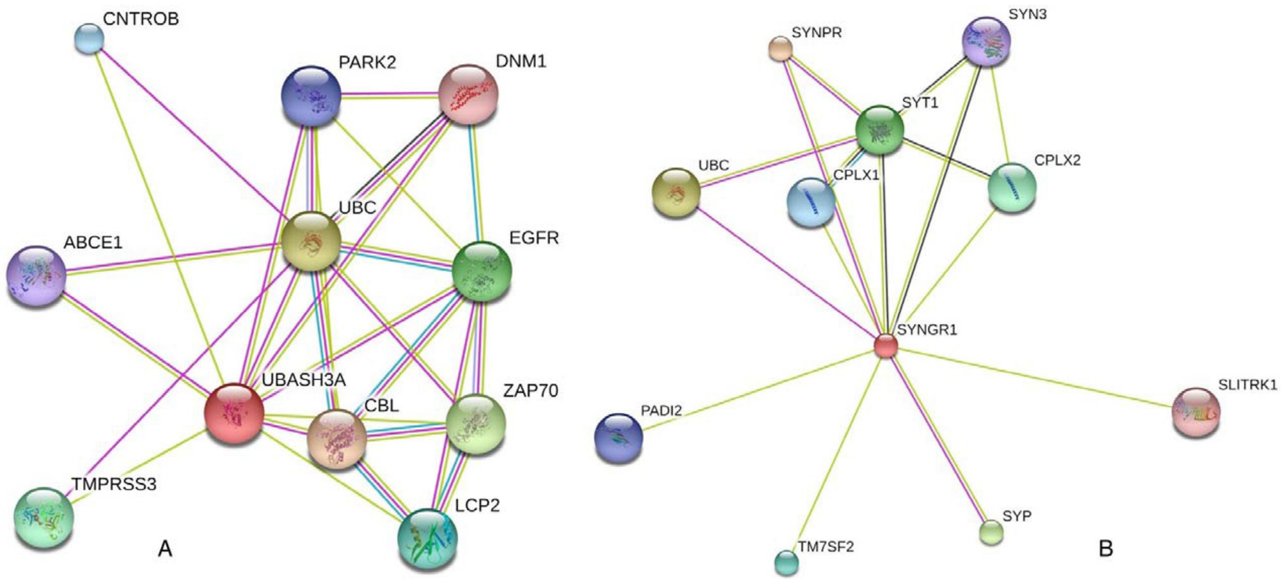

Supplementary Figure 1: (**A**) Protein-protein interaction plot for gene *UBASH3A*. (**B**) Protein-protein interaction plot for gene *SYNGR1*.

A

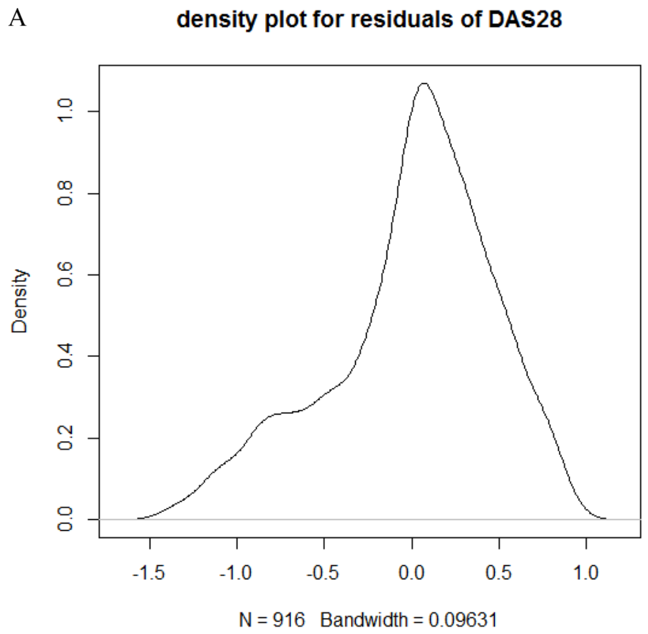

B

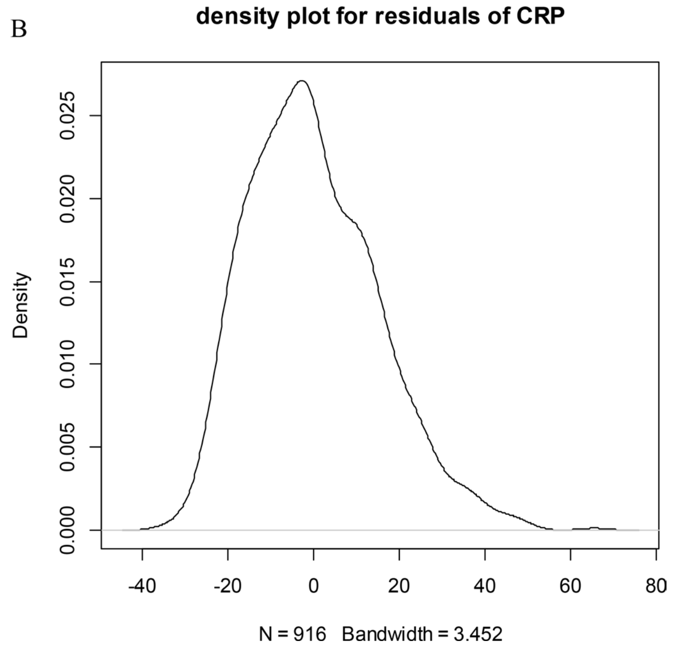

Supplementary Figure 2: (**A**) Density plot for residuals of DAS28 for rs1893592. (**B**) Density plot for residuals of CRP for rs1893592.
